# Successful Pregnancy Outcome after Laparoscopic Cerclage in a Patient with Cervicovaginal Fistula

**DOI:** 10.1155/2015/784025

**Published:** 2015-10-25

**Authors:** Giovanni Zanconato, Valentino Bergamini, Silvia Baggio, Elena Cavaliere, Massimo Franchi

**Affiliations:** ^1^Department of Surgical, Odontostomatological and Maternal and Child Sciences, University of Verona, 37134 Verona, Italy; ^2^Azienda Ospedaliera Universitaria Integrata of Verona, 37134 Verona, Italy

## Abstract

Obstetric fistula usually originates from obstructed labor or, less often, from invasive maneuvers on the genital tract or the pregnant uterus. Overall, it is a rare finding in the obstetric practice of high income countries. In this report we describe the case of a successful term pregnancy in a patient with a history of recurrent late miscarriage due to a large cervical fistula of traumatic origin, connecting the uterine cavity and the posterior vaginal fornix. A combined approach of laparoscopic cerclage and transvaginal fistula repair effectively restored cervical competence and created the conditions for a viable birth in a subsequent pregnancy. This unusual cause of cervical incompetence may be included in the indications which benefit from an abdominal cerclage carried out as a minimally invasive procedure in the nonpregnant state.

## 1. Introduction

Laparoscopic cerclage is a relatively new procedure which has limited application among the variety of therapeutic options used to prevent preterm birth [1, 2]. Just like abdominal cerclage via laparotomy, it is considered a second-line treatment for cervical incompetence whenever vaginal cerclage is inappropriate or has failed. Primary indications are a congenitally short or absent cervix and previous cervical surgery [3]. Placement of the cerclage may be carried out during pregnancy or as an interval procedure. Several studies have concluded that the laparoscopic procedure is feasible and effective and compares favourably with the “traditional” abdominal approach having the advantage of a substantial reduction in recovery time and a significant shorter period of hospitalization [3–6].

We report the case of a successful term pregnancy obtained after positioning a laparoscopic cerclage in a patient with a history of recurrent mid-trimester miscarriage for whom all previous attempts of repair had failed.

## 2. Case Presentation

**Figure 1:**
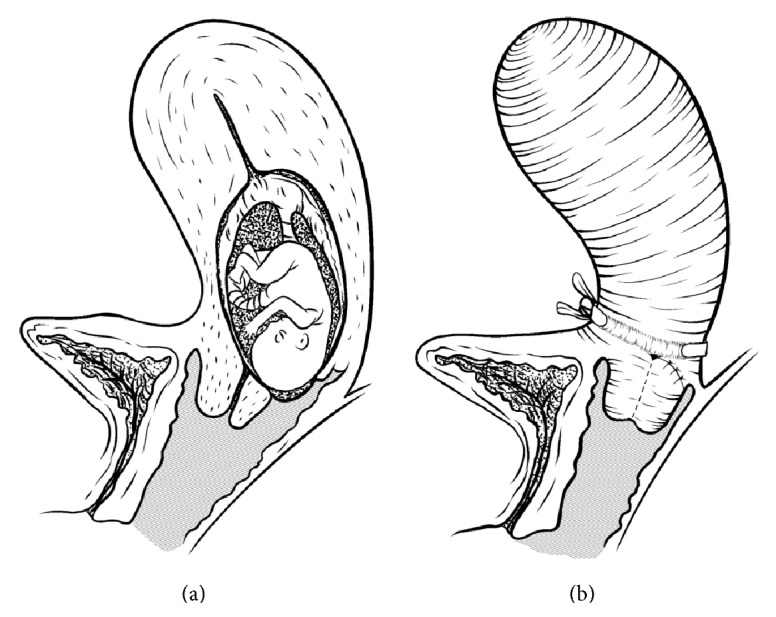
(a) Gestational sac bulging through fistula; (b) sutured fistula and cerclage.

A 30-year-old nulliparous woman of Nigerian origin was admitted in the Obstetrical Department of the University Hospital of Verona with pPROM and active uterine contractions at 23 weeks' gestation. Previous medical history was unremarkable except for an illegal abortion induced by means of an instrumental traditional method in the country of origin some years before. A liveborn male fetus of 560 g was delivered but died in the Intensive Care Neonatal Unit in the early postnatal period. A transvaginal examination in the delivery room showed a 4 cm wide uterovaginal fistula: the supracervical lesion in close proximity of the internal cervical os and connecting the uterine cavity with the posterior fornix was repaired right after the 3rd stage of labor. A few months later the same patient came again under our observation with the clinical presentation of a late miscarriage at 18 weeks due to recurrence of the fistula. The former suture had failed to hold and the intact gestational sac was bulging through the uterovaginal opening (Figure 1(a)) with spontaneous expulsion occurring shortly after. In order to increase chances of a successful repair it was decided to differ a new hysteroplasty to a later time. The patient went through a 3-month course of continuous combined low-dose hormonal treatment and was then scheduled for surgery: the procedure consisted of a transvaginal fistula repair associated with laparoscopic cerclage. The placement of the cerclage was performed under general anesthesia. After dissecting the uterovesical space, a 5 mm nonabsorbable polyester ligature with double blunt needles was introduced into the abdominal cavity. The fiber suture was placed by passing each needle from posterior to anterior, medial to the uterine vessels, just over the internal cervical os bilaterally. Needles were then cut and removed and the tape was tied tightly around the cervix with intracorporeal knots (Figure 1(b)). The uterovesical peritoneum was then reapproximated to cover the knot. One year later the patient started a new pregnancy which proceeded uncomplicated until a caesarean section was performed at 37 weeks' gestation and a healthy 3,000 g boy was delivered. Intraoperative examination of the lower segment found no sign of dehiscence of the posterior uterine wall. The cerclage was left in situ in view of the patient's desire for other children. The posterior vaginal fornix was intact as well, except for a slight mucosal retraction, the result of the previous repair.

## 3. Discussion

This is a rare case of posttraumatic cervicovaginal fistula affecting a recently immigrated sub-Saharan African woman. Past history of the patient, that is, invasive maneuvers associated with a previous illegal abortion, well explained the lesion observed and the recurrent late miscarriages.

A previous attempt to regain the uterine integrity by simply suturing the fistula vaginally had proved unsuccessful. It was deemed necessary to combine a second fistula repair with a cerclage which would strengthen the lower segment. It was assumed that the cerclage would help withstand the direct pressure exerted by the pregnancy downward over the weakened cervix and the adjoining injured area.

Accordingly our choice was the transabdominal approach since it allowed a higher placement of the ligature at the level of the cervical internal os. The decision to use the laparoscopic approach was based on the evidence that this technique is as effective as a cerclage placed via laparotomy [6] but has the advantages of a minimally invasive surgical procedure, such as reduced blood loss and postoperative pain, fewer adhesions, shorter hospital stay, and faster recovery. Also the importance of avoiding a laparotomy to place the cerclage in the first instance is obvious, considering that mode of delivery will necessarily be a cesarean.

It should be acknowledged however that, given the scarcity of cases in need of a transabdominal cerclage, whichever the approach, the intervention is seldom performed and the experience of the surgical team is limited. This fact justifies, in our opinion, the decision to place the cerclage preferably in the preconception period, as an interval procedure. Decreased fetomaternal risk, easier manipulation and exposure, and less bleeding are all associated with the procedure in the nonpregnant state. Because of cervical softening, the laparoscopic approach during pregnancy increases the risk of bleeding, due to a diminished ability to distinguish between the isthmus and adjacent vessels by tactile feedback [2, 7].

A disadvantage of the procedure, as any interval procedure, is that pregnancy may either never occur or result in early loss. In view of this possible outcome the placement of a cervical dilator has been suggested when the cerclage is tied; by doing so a suction curettage may eventually be performed since the cerclage will not cause a cervical stenosis [2, 5].

Results from published studies are reassuring in terms of pregnancy outcome after laparoscopic cerclage: chances to conceive following abdominal cerclage do not seem to be impaired as we observed in our patient who got pregnant within the year. Fetal survival rates following laparoscopic cerclage placement are reported in the range of 76 to 100% and compare favourably with the traditional approach by laparotomy with a substantial reduction in morbidity [7–10].

In conclusion, the combined approach of laparoscopic cerclage and transvaginal fistula repair effectively restored the conditions for a viable birth in a subsequent pregnancy. This rather unique cause of recurrent late miscarriage may be included in the indications which benefit from an abdominal cerclage carried out as a minimally invasive procedure in the nonpregnant state.
